# Crystal structure study of a cobaltoan dolomite from Kolwezi, Democratic Republic of Congo

**DOI:** 10.1107/S2056989015003126

**Published:** 2015-02-21

**Authors:** Natale Perchiazzi

**Affiliations:** a Earth Sciences Department, Pisa University, Via S. Maria 53, I-56126, Pisa, Italy

**Keywords:** crystal structure, dolomite, cobaltoan, Kolwezi

## Abstract

A structural study has been undertaken on a cobaltoan dolomite, with chemical formula CaMg_0.83_Co_0.17_(CO_3_)_2_ (cal­cium magnesium cobalt dicarbonate), from Kolwezi, Democratic Republic of Congo. Pale-pink euhedral cobaltoan dolomite was associated with kolwezite [(Cu_1.33_Co_0.67_)(CO_3_)(OH)_2_] and cobaltoan malachite [(Cu,Co)_2_(CO_3_)(OH)_2_]. A crystal with a Co:Mg ratio of 1:5.6 (SEM/EDAX measurement), twinned on (11 -2 0) was used for crystal structural refinement. The refinement of the structural model of Reeder & Wenk [*Am. Mineral.* (1983 ▸), **68**, 769–776; Ca at site 3*a* with site symmetry -3; Mg site at site 3*b* with site symmetry -3; C at site 6*c* with site symmetry 3; O at site 18*f* with site symmetry 1] showed that Co is totally incorporated in the Mg site, with refined occupancy Mg_0.83_Co_0.17_, which compares with Mg_0.85_Co_0.15_ from chemical data. The Co substitution reflects in the expansion of the cell volume, with a pronounced increasing of the *c* cell parameter.

## Related literature   

For general background, see: Barton *et al.* (2015 ▸); Pertlik (1986 ▸). For isotypic structures, see: Reeder & Wenk (1983 ▸). For kolwezite, see: Deliens & Piret (1980 ▸).

## Experimental   

### Crystal data  

CaMg_0.83_Co_0.17_(CO_3_)_2_
*M* *_r_* = 190.38
Trigonal, 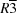

*a* = 4.8158 (1) Å
*c* = 16.0488 (6) Å
*V* = 322.34 (2) Å^3^
*Z* = 3
Mo *K*α radiation
μ = 2.17 mm^−1^
*T* = 295 K
0.20 × 0.15 × 0.12 mm

### Data collection  

Bruker SMART Breeze CCD diffractometer
Absorption correction: multi-scan (*SADABS*; Bruker, 2008 ▸) *T*_min_ = 0.621, *T*_max_ = 0.746
738 measured reflections
258 independent reflections
257 reflections with *I* > 2σ(*I*)
*R*_int_ = 0.010

### Refinement  

*R*[*F* ^2^ > 2σ(*F* ^2^)] = 0.019
*wR*(*F* ^2^) = 0.059
*S* = 0.96
258 reflections
20 parameters
1 restraint
Δρ_max_ = 0.46 e Å^−3^
Δρ_min_ = −0.32 e Å^−3^

### 

Data collection: *APEX2* (Bruker, 2008 ▸); cell refinement: *SAINT* (Bruker, 2008 ▸); data reduction: *SAINT*; program(s) used to solve structure: *SHELXS2014* (Sheldrick, 2008 ▸); program(s) used to refine structure: *SHELXL2014* (Sheldrick, 2008 ▸, 2015 ▸) and *WinGX* (Farrugia, 2012 ▸); molecular graphics: *CrystalMaker* (CrystalMaker, 2010 ▸); software used to prepare material for publication: *publCIF* (Westrip, 2010 ▸).

## Supplementary Material

Crystal structure: contains datablock(s) I, New_Global_Publ_Block. DOI: 10.1107/S2056989015003126/br2247sup1.cif

Structure factors: contains datablock(s) I. DOI: 10.1107/S2056989015003126/br2247Isup2.hkl

**Fig. 1.**
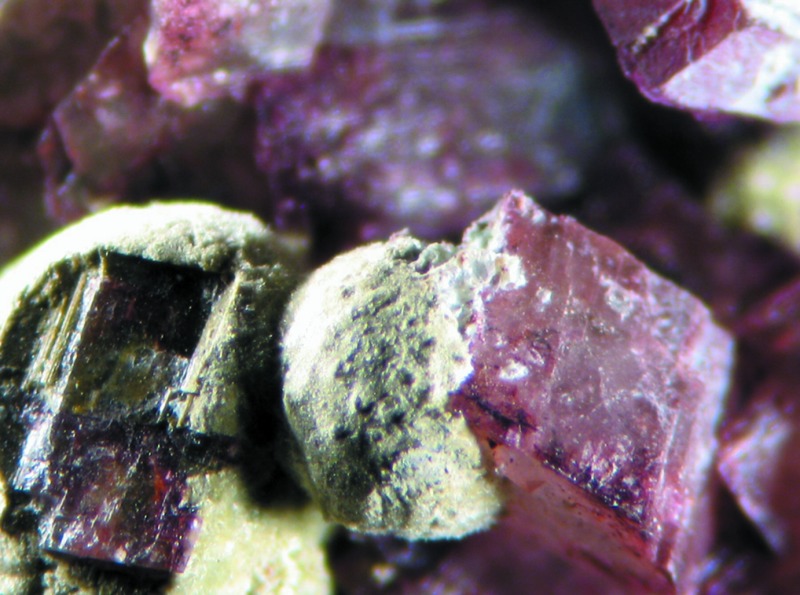
Micro photograph of the cobaltoan dolomite specimen, where pale pink cobaltoan dolomite is associated with pale green cobaltoan malachite.

**Fig. 2.**
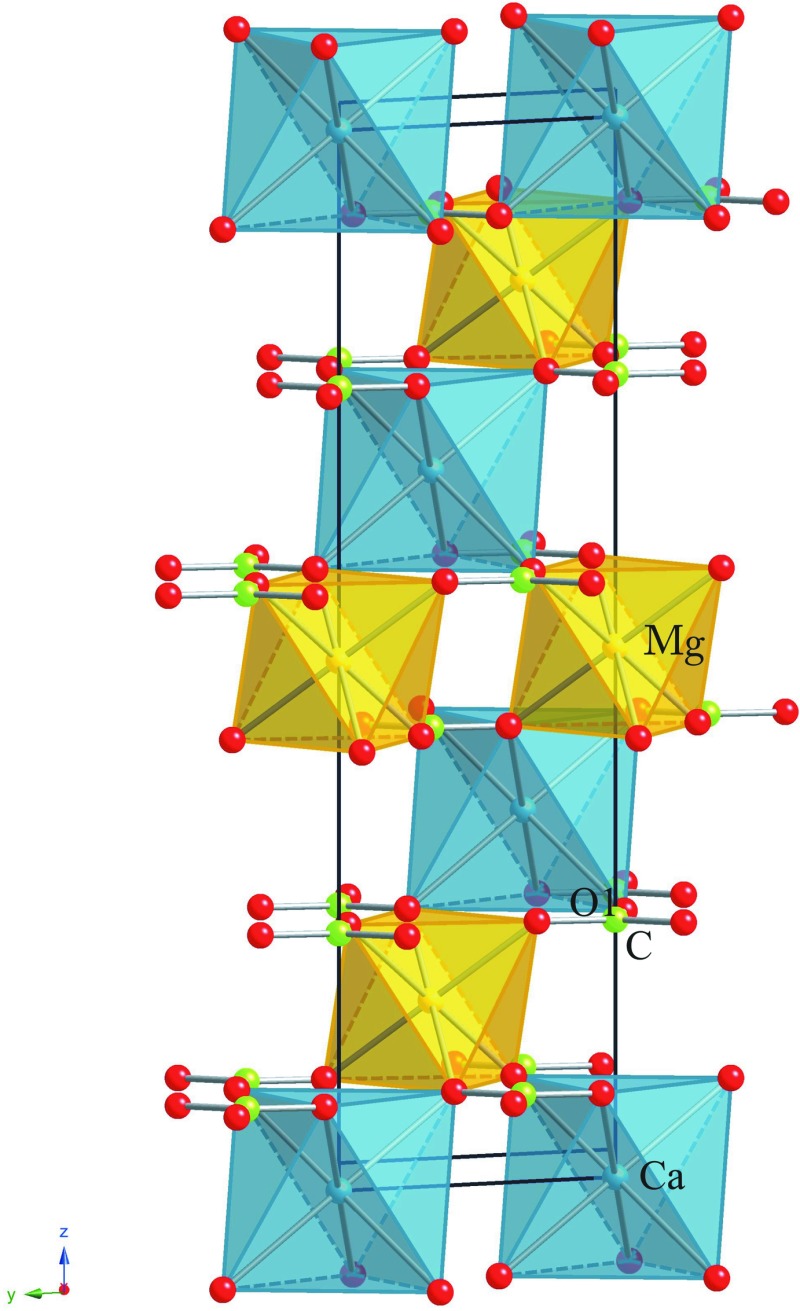
The crystal structure of cobaltoan dolomite, in a projection along [100], slightly tilted by 5° about along the x Cartesian rotation axis. Ca-centered octahedra are cyan, whereas Mg-centered octahedra are yellow; carbon and oxygen atoms are represented as green and red spheres, respectively.

. DOI: 10.1107/S2056989015003126/br2247fig1.tif
Micro photograph of the cobaltoan dolomite specimen, where pale pink cobaltoan dolomite is associated with pale green cobaltoan malachite.

x . DOI: 10.1107/S2056989015003126/br2247fig2.tif
The crystal structure of cobaltoan dolomite, in a projection along [100], slightly tilted by 5° about along the *x* Cartesian rotation axis. Ca-centered octa­hedra are cyan, whereas Mg-centered octa­hedra are yellow; carbon and oxygen atoms are represented as green and red spheres, respectively.

**Fig. 3.**
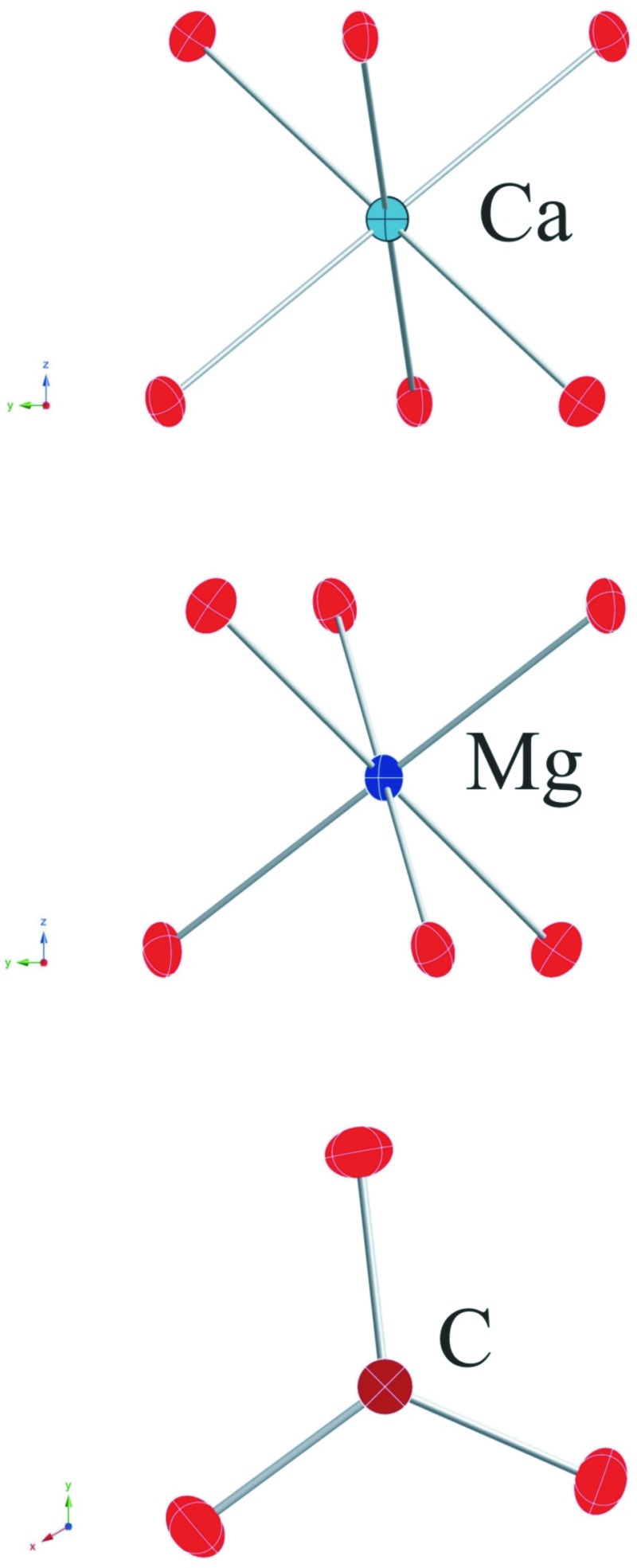
Coordination polyhedra in cobaltoan dolomite. Displacement ellipsoids are drawn at the 50% probability.

. DOI: 10.1107/S2056989015003126/br2247fig3.tif
Coordination polyhedra in cobaltoan dolomite. Displacement ellipsoids are drawn at the 50% probability.

CCDC reference: 1049359

Additional supporting information:  crystallographic information; 3D view; checkCIF report

## Figures and Tables

**Table 1:** Selected bond lengths ()

Ca1O1^i^	2.3833(10)
(Mg1/Co1)O1^ii^	2.0923(9)
C1O1	1.2853(9)

Symmetry codes: (i) 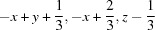; (ii) 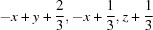.

## supplementary crystallographic information

### S1. Synthesis and crystallization

Cobaltoan dolomite was picked from a kolwezite sample from Kolwezi (inventory number RC 3987) kindly provided us by H. Goethals, Royal Belgian Institute for Natural Sciences, Brussels. Pale pink euhedral cobaltoan dolomite was associated with kolwezite and cobaltoan malachite. All these minerals occur in the supergene zones of Cu—Co sulfide ore deposits,originating from the alteration of primary sulphides such as carrollite,Cu(Co,Ni)_2_As_4_

### S2. Refinement

During the refinement, the twinning according to the (1120) common law was detected and accounted for, with a refined BASF parameter of 0.798. The sum of Co and Mg occupancies in Mg site was constrained to be equal to 1, no other constraint was applied.

### Crystal data

**Table:**

CaMg_0.83_Co_0.17_(CO_3_)_2_	*F*(000) = 284
*M**_r_* = 190.38	*D*_x_ = 2.930 Mg m^−^^3^
Trigonal, *R*3	Mo *K*α radiation, λ = 0.71073 Å
*a* = 4.8158 (1) Å	µ = 2.17 mm^−^^1^
*c* = 16.0488 (6) Å	*T* = 295 K
*V* = 322.34 (2) Å^3^	Cleavage rhombohedron, pale pink
*Z* = 3	0.2 × 0.15 × 0.12 mm

### Data collection

**Table:**

Bruker SMART Breeze CCD diffractometer	257 reflections with *I* > 2σ(*I*)
ω scans	*R*_int_ = 0.010
Absorption correction: multi-scan (*SADABS*; Bruker, 2008)	θ_max_ = 32.4°, θ_min_ = 3.8°
*T*_min_ = 0.621, *T*_max_ = 0.746	*h* = −7→3
258 measured reflections	*k* = 0→7
738 independent reflections	*l* = −23→23

### Refinement

**Table:**

Refinement on *F*^2^	20 parameters
Least-squares matrix: full	1 restraint
*R*[*F*^2^ > 2σ(*F*^2^)] = 0.019	*w* = 1/[σ^2^(*F*_o_^2^) + (0.0438*P*)^2^ + 0.562*P*] where *P* = (*F*_o_^2^ + 2*F*_c_^2^)/3
*wR*(*F*^2^) = 0.059	(Δ/σ)_max_ < 0.001
*S* = 0.96	Δρ_max_ = 0.46 e Å^−^^3^
258 reflections	Δρ_min_ = −0.32 e Å^−^^3^

### Special details

**Table:**

Geometry. All e.s.d.'s (except the e.s.d. in the dihedral angle between two l.s. planes) are estimated using the full covariance matrix. The cell e.s.d.'s are taken into account individually in the estimation of e.s.d.'s in distances, angles and torsion angles; correlations between e.s.d.'s in cell parameters are only used when they are defined by crystal symmetry. An approximate (isotropic) treatment of cell e.s.d.'s is used for estimating e.s.d.'s involving l.s. planes.
Refinement. Refined as a 2-component twin.

### Fractional atomic coordinates and isotropic or equivalent isotropic displacement parameters (Å^2^)

**Table:**

	*x*	*y*	*z*	*U*_iso_*/*U*_eq_	Occ. (<1)
Ca1	0.0000	0.0000	0.0000	0.01249 (17)	
Mg1	0.0000	0.0000	0.5000	0.0104 (3)	0.828 (4)
Co1	0.0000	0.0000	0.5000	0.0104 (3)	0.172 (4)
C1	0.0000	0.0000	0.24297 (12)	0.0106 (4)	
O1	0.2482 (2)	−0.0341 (2)	0.24403 (6)	0.0143 (2)	

### Atomic displacement parameters (Å^2^)

**Table:**

	*U*^11^	*U*^22^	*U*^33^	*U*^12^	*U*^13^	*U*^23^
Ca1	0.0121 (2)	0.0121 (2)	0.0133 (3)	0.00605 (10)	0.000	0.000
Mg1	0.0091 (3)	0.0091 (3)	0.0130 (4)	0.00456 (15)	0.000	0.000
Co1	0.0091 (3)	0.0091 (3)	0.0130 (4)	0.00456 (15)	0.000	0.000
C1	0.0103 (5)	0.0103 (5)	0.0112 (8)	0.0051 (3)	0.000	0.000
O1	0.0117 (4)	0.0156 (4)	0.0183 (4)	0.0088 (3)	−0.0024 (3)	−0.0033 (3)

### Geometric parameters (Å, º)

**Table:**

Ca1—O1^i^	2.3833 (10)	Mg1—O1^ix^	2.0923 (9)
Ca1—O1^ii^	2.3833 (10)	Mg1—O1^x^	2.0923 (9)
Ca1—O1^iii^	2.3833 (10)	Mg1—O1^xi^	2.0923 (9)
Ca1—O1^iv^	2.3833 (10)	Mg1—O1^xii^	2.0923 (9)
Ca1—O1^v^	2.3833 (10)	C1—O1	1.2853 (9)
Ca1—O1^vi^	2.3833 (10)	C1—O1^xiii^	1.2853 (9)
Mg1—O1^vii^	2.0923 (9)	C1—O1^xiv^	1.2853 (9)
Mg1—O1^viii^	2.0923 (9)	C1—Ca1^xv^	3.1359 (9)
			
O1^i^—Ca1—O1^ii^	180.00 (5)	O1^viii^—Mg1—O1^ix^	91.62 (4)
O1^i^—Ca1—O1^iii^	92.43 (3)	O1^vii^—Mg1—O1^x^	91.62 (4)
O1^ii^—Ca1—O1^iii^	87.57 (3)	O1^viii^—Mg1—O1^x^	88.38 (4)
O1^i^—Ca1—O1^iv^	87.57 (3)	O1^ix^—Mg1—O1^x^	180.0
O1^ii^—Ca1—O1^iv^	92.43 (3)	O1^vii^—Mg1—O1^xi^	91.62 (4)
O1^iii^—Ca1—O1^iv^	180.00 (4)	O1^viii^—Mg1—O1^xi^	88.38 (4)
O1^i^—Ca1—O1^v^	87.57 (3)	O1^ix^—Mg1—O1^xi^	91.62 (4)
O1^ii^—Ca1—O1^v^	92.43 (3)	O1^x^—Mg1—O1^xi^	88.38 (4)
O1^iii^—Ca1—O1^v^	92.43 (3)	O1^vii^—Mg1—O1^xii^	88.38 (4)
O1^iv^—Ca1—O1^v^	87.57 (3)	O1^viii^—Mg1—O1^xii^	91.62 (4)
O1^i^—Ca1—O1^vi^	92.43 (3)	O1^ix^—Mg1—O1^xii^	88.38 (4)
O1^ii^—Ca1—O1^vi^	87.57 (3)	O1^x^—Mg1—O1^xii^	91.62 (4)
O1^iii^—Ca1—O1^vi^	87.57 (3)	O1^xi^—Mg1—O1^xii^	180.00 (4)
O1^iv^—Ca1—O1^vi^	92.43 (3)	O1—C1—O1^xiii^	119.984 (5)
O1^v^—Ca1—O1^vi^	180.00 (8)	O1—C1—O1^xiv^	119.983 (5)
O1^vii^—Mg1—O1^viii^	180.0	O1^xiii^—C1—O1^xiv^	119.981 (5)
O1^vii^—Mg1—O1^ix^	88.38 (4)		

Symmetry codes: (i) −*x*+*y*+1/3, −*x*+2/3, *z*−1/3; (ii) *x*−*y*−1/3, *x*−2/3, −*z*+1/3; (iii) −*x*+2/3, −*y*+1/3, −*z*+1/3; (iv) *x*−2/3, *y*−1/3, *z*−1/3; (v) −*y*+1/3, *x*−*y*−1/3, *z*−1/3; (vi) *y*−1/3, −*x*+*y*+1/3, −*z*+1/3; (vii) −*x*+*y*+2/3, −*x*+1/3, *z*+1/3; (viii) *x*−*y*−2/3, *x*−1/3, −*z*+2/3; (ix) −*y*−1/3, *x*−*y*−2/3, *z*+1/3; (x) *y*+1/3, −*x*+*y*+2/3, −*z*+2/3; (xi) −*x*+1/3, −*y*−1/3, −*z*+2/3; (xii) *x*−1/3, *y*+1/3, *z*+1/3; (xiii) −*x*+*y*, −*x*, *z*; (xiv) −*y*, *x*−*y*, *z*; (xv) *x*+2/3, *y*+1/3, *z*+1/3.
