# Supplementary figures and images for: Crystal structure study of a cobaltoan dolomite from Kolwezi, Democratic Republic of Congo
Source: Acta Crystallogr E Crystallogr Commun. 2015 Feb 21;71(Pt 3):i3. doi: 10.1107/S2056989015003126 (PMC4350686; doi:10.1107/S2056989015003126)

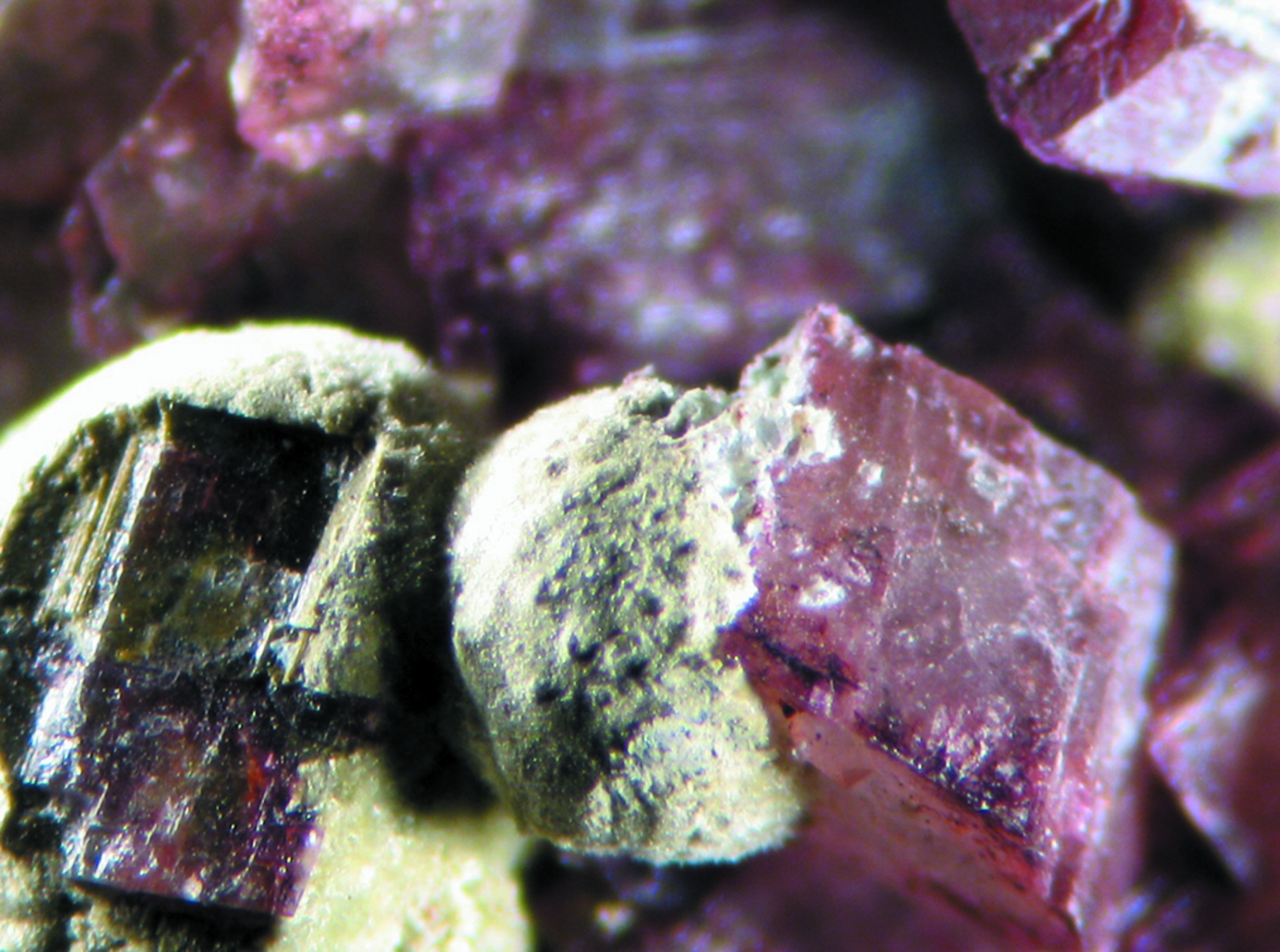

Supplement: Supplementary file 3 [file e-71-000i3-fig1.tif]

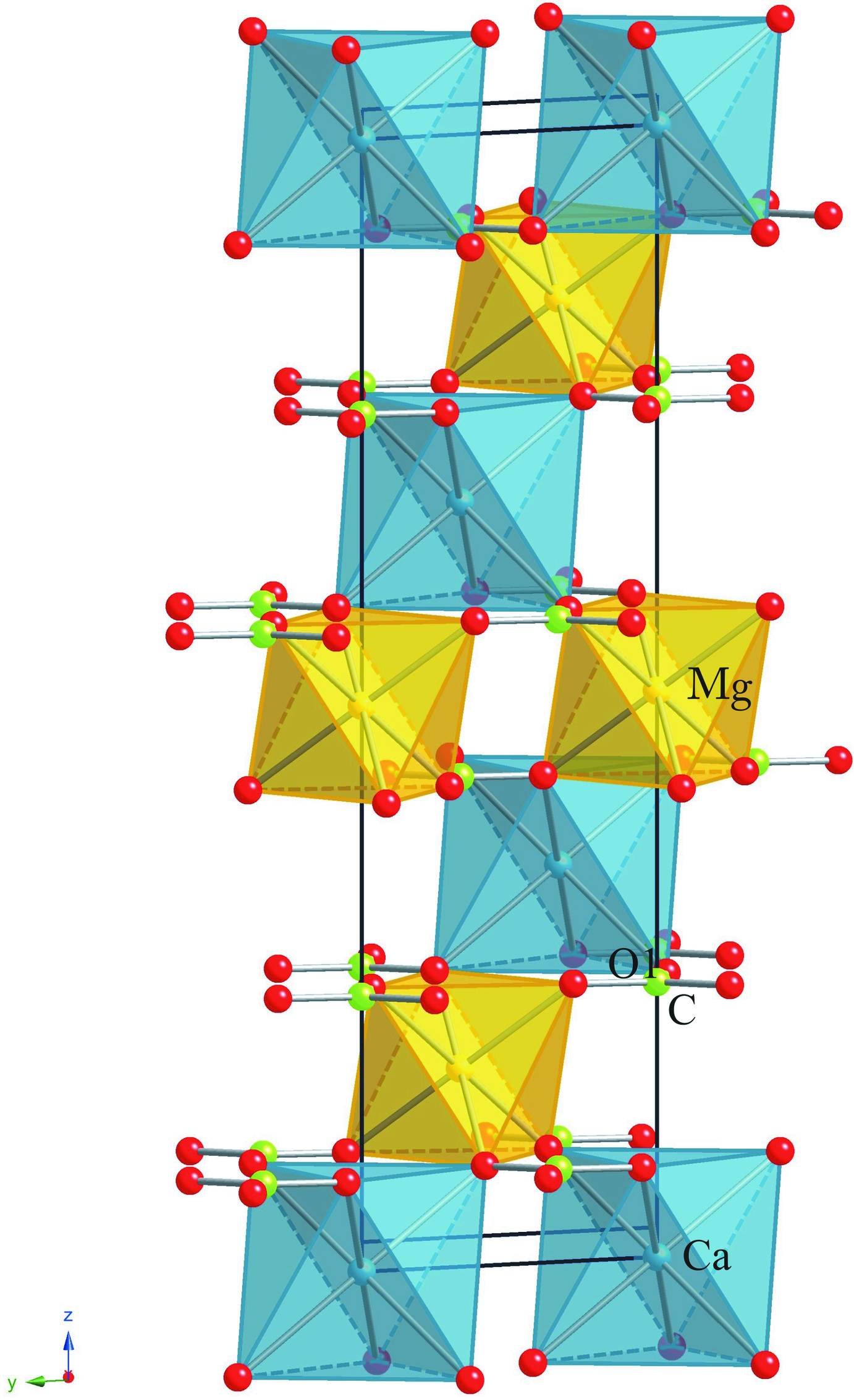

Supplement: Supplementary file 4 [file e-71-000i3-fig2.tif]

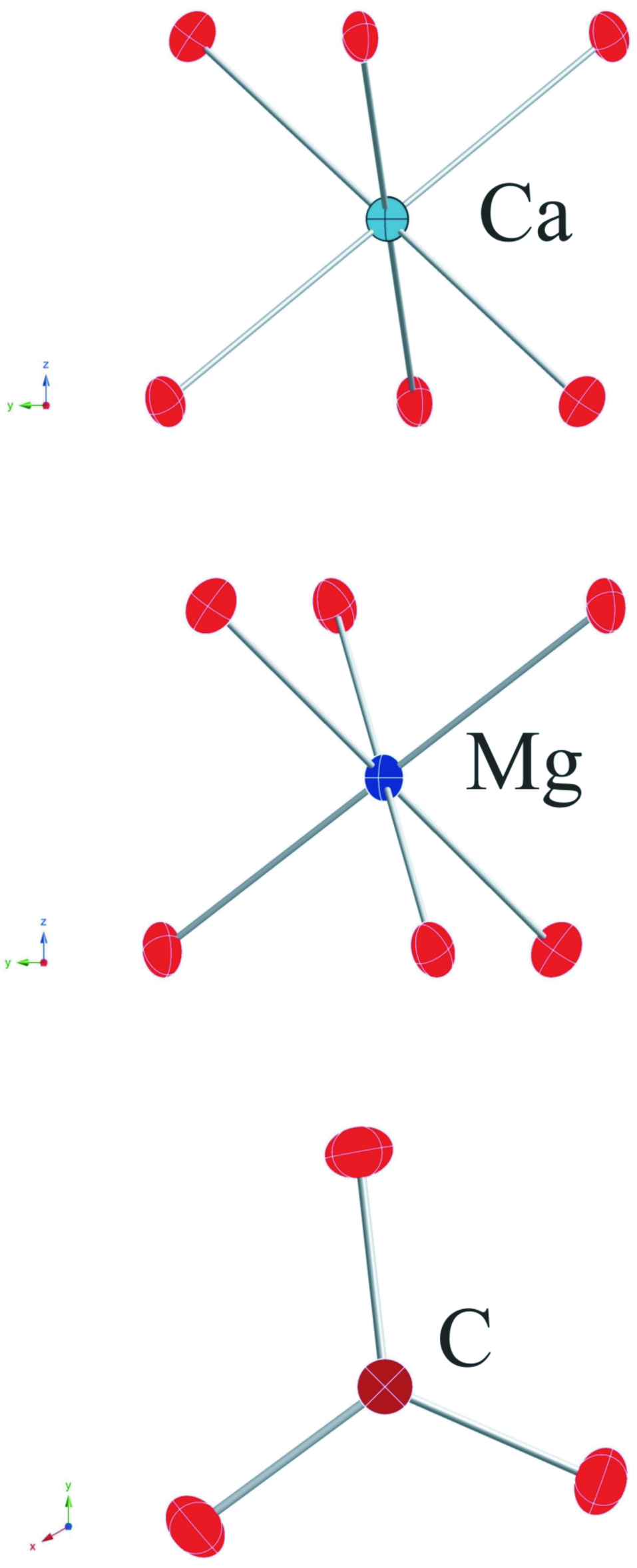

Supplement: Supplementary file 5 [file e-71-000i3-fig3.tif]
